# Novel Enhanced Recovery After Surgery Pathway Reduces Length of Stay and Postoperative Opioid Usage in Adolescent Idiopathic Scoliosis Patients Undergoing Posterior Spinal Fusion

**DOI:** 10.7759/cureus.43079

**Published:** 2023-08-07

**Authors:** Kristen Spisak, Matthew D Thomas, Zachary J Sirois, Alvin Jones, Lucinda Brown, Andrew W Froehle, Michael Albert

**Affiliations:** 1 Anesthesia, Dayton Children's Hospital, Dayton, USA; 2 Orthopedic Surgery, Dayton Children's Hospital, Dayton, USA; 3 Orthopedic Surgery, Mount Carmel Health System, Columbus, USA; 4 Nursing, Dayton Children's Hospital, Dayton, USA; 5 Orthopedic Surgery, Miami Valley Hospital, Dayton, USA

**Keywords:** pain pathway, recovery, scoliosis, opioid, length of stay

## Abstract

Purpose: The goal of this study was to compare our institution’s recently implemented enhanced recovery after surgery (ERAS) protocol to previous post-operative management for adolescent idiopathic scoliosis patients undergoing posterior spinal fusion, specifically assessing length of stay, opioid consumption, and pain scores.

Methods: This is a retrospective analysis that compares the length of stay, opioid consumption, and pain scores of patients undergoing posterior spinal fusion for adolescent idiopathic scoliosis. Patients were analyzed prior to the implementation of our ERAS protocol, deemed the traditional pain pathway (TPP), to those who underwent the ERAS pathway. All patients undergoing posterior spinal fusion for adolescent idiopathic scoliosis were included. Patients were excluded if they weighed less than 40kg, had significant comorbidities, or had non-idiopathic causes of scoliosis.

Results: We examined 22 patients in the TPP cohort and 20 in the ERAS cohort. Length of stay in the ERAS cohort was significantly reduced compared to the TPP by 1.7 days (P<0.01). Overall opioid consumption was also significantly reduced in the ERAS with 1.4 ± 0.7 morphine equivalents (ME)/kg compared to the TPP 2.4 ± 1.1 ME/kg (P < 0.01). We found no difference in pain scores between the two groups.

Conclusion: Implementation of an ERAS pathway at our institution significantly reduced length of stay and opioid consumption in adolescent idiopathic scoliosis patients undergoing posterior spinal fusion. These outcomes reduce morbidity and costs associated with posterior spinal fusion and provide an overall improvement in the quality of care for our patients.

## Introduction

The postoperative period for adolescent idiopathic scoliosis (AIS) patients undergoing posterior spinal fusion (PSF) is fraught with challenges, including adequate postoperative pain control and prolonged hospitalization. Inadequate postoperative pain control can contribute to prolonged hospital stays and even intensive care unit (ICU) admissions. While intravenous opioid medications are the mainstay of postoperative pain management for PSF patients, other techniques include epidural analgesics, intrathecal opioids, systemic ketamine, and gabapentinoids [1]. Despite varying methods for pain management, patients’ pain often remains difficult to control.

Enhanced recovery after surgery (ERAS) protocols were originally developed to improve overall outcomes in surgical patients [2]. In the early 2000s, colorectal surgeons first implemented ERAS protocols, and their patients experienced reduced morbidity and decreased length of hospital stay (LOS) [3]. One aspect of ERAS emphasizes scheduled multimodal pain medication regimens that allow patients to achieve earlier postoperative milestones, resulting in a shorter LOS [4]. Orthopedic surgeons have more recently adopted ERAS protocols for adult patients undergoing total joint arthroplasties and spine operations, which has led to a significant reduction in LOS, postoperative pain, and overall cost [5,6]. Although ERAS pathways are now commonplace in the adult realm, they remain relatively understudied in the pediatric surgical population [7,8].

Studies examining ERAS outcomes in AIS patients undergoing PSF are few [9-13]. The goal of this study was to compare our institution’s recently implemented ERAS protocol to previous post-operative management for AIS patients undergoing PSF, specifically assessing length of stay, opioid consumption, and pain scores.

## Materials and methods

In this study, we assessed the efficacy of a newly implemented ERAS protocol for patients with AIS undergoing PSF. Primary outcomes included LOS, opioid usage, and pain scores. All patients included in this study had a PSF performed by one of two orthopedic surgeons from December 2013 to April 2018. All patients who underwent PSF for AIS were included in the study. Patients were excluded if they weighed less than 40 kg, had significant comorbidities, or had non-idiopathic causes of scoliosis. The traditional pain pathway (TPP) cohort included patients who had their operations between December 2013 and April 2017. The ERAS cohort included all patients from May 2017 to April 2018, after the implementation of the ERAS protocol. This change served as a surrogate for intervention in our study allowing us to analyze the differences between these two groups. We collected all data retrospectively from our institution’s electronic medical records and recorded the LOS from the date of surgery until discharged from the hospital. Postoperative opioid usage was recorded and all medications were converted to morphine-equivalents (ME). ME per kg (ME/kg) was then calculated. Nurse charting of pain levels using the visual analog scale (VAS) was reviewed and the daily average and highest pain score was recorded for each patient. We also reviewed each group for any potential complications including urinary retention, neurologic deficits, local wound pathology, prolonged ventilation, or return to trip to the operating room within three months. Our institutional review board reviewed and approved this study.

Traditional pain pathway (TPP)

The TPP included surgeon-administered intraoperative intrathecal morphine, a first postoperative night stay in the pediatric intensive care unit (PICU), and highly variable postoperative pain medication administration. Without a standardized protocol, much of the post-operative pain management was left to the discretion of different medical teams. Teams that managed postoperative pain during the TTP varied between the pediatric medicine team and the primary surgical team without consistency. Furthermore, members of these teams frequently changed personnel which resulted in a wide variety of opioid and non-opioid medications utilized. These included morphine, oxycodone, and hydromorphone. Providers proved a range of frequency and dosages of pain medication administration ranging from patient-controlled to PRN.

ERAS pathway

Our ERAS protocol emphasized multimodal pain control. The acute pain service (APS) managed all postoperative pain medications in order to decrease variability in treatment. Preoperatively patients received gabapentin, celecoxib, and midazolam. Intraoperatively, they received total intravenous anesthetic of propofol, remifentanil, and ketamine, while receiving methadone, dexamethasone, ondansetron, acetaminophen, and methocarbamol for pain control. Since the use of intrathecal morphine was abandoned, patients no longer required monitoring in the PICU on the first night postoperatively. They went from the post-anesthesia care unit (PACU) to the general post-surgical floor. On postoperative day 0 (POD0), patients were placed on hydromorphone, methadone, acetaminophen, ketorolac, methocarbamol, ondansetron, diazepam, and a bowel regimen. On POD1, gabapentin and oxycodone were added. Ketorolac and diazepam were discontinued and ibuprofen was started on POD2. On the morning of POD3, hydromorphone was discontinued and patients remained on an oral pain regimen for the remainder of their stay comprising oxycodone, acetaminophen, ibuprofen, methocarbamol, ondansetron, gabapentin, and a bowel regimen. See Appendix - Figure 4, for the full summary of the ERAS protocol. Additionally, our ERAS pathway emphasized the achievement of early postoperative milestones, including early transition from IV to oral pain medications and prompt return to activities of daily living.

Patient selection

**Figure 1:**
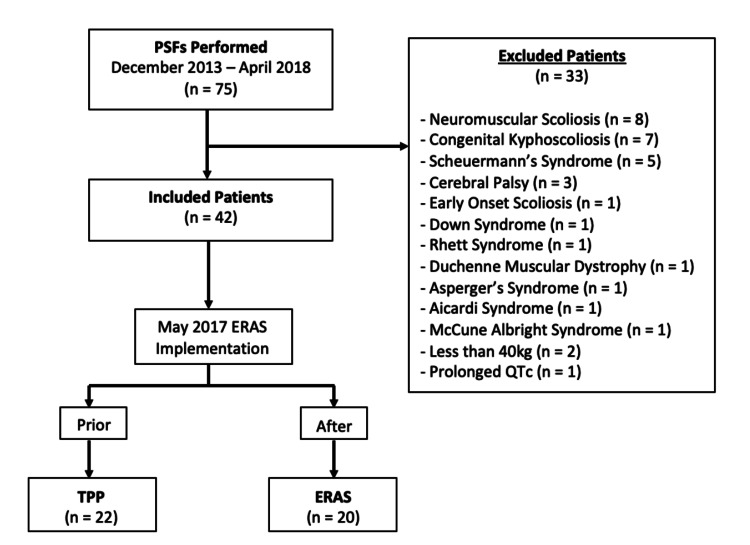
Consecutive patients meeting inclusion criteria were reviewed with 33 meeting exclusion criteria, and the remaining 42 were further divided by which pain protocol was used during their treatment. PSF: posterior spinal fusion, ERAS: enhanced recovery after surgery, TPP: traditional pain pathway

We identified all patients undergoing PSF performed by two different surgeons from December 2013 to April 2018 (n = 75). Of the cases reviewed, we excluded 33 patients due to non-idiopathic causes of scoliosis as shown in Figure 1.

Additionally, we excluded patients who weighed less than 40kg (n = 2) and a patient with pre-operative prolonged QTc because the medications included in the ERAS pathway were contraindicated. Of the remaining patients (n = 42), the TPP group included all surgeries prior to May 2017 (n = 22) and the ERAS group included those after the implementation of the new protocol (n = 20).

Statistical analysis

All statistical analysis was performed in Statistical Analysis Software (SAS) 9.4 (SAS Institute, Cary, NC), with significance set to α=0.05. Baseline preoperative characteristics of the two groups were compared using Wilcoxon-Mann-Whitney tests for interval data, and Fisher’s exact test for nominal frequency data. Postoperative differences in LOS, total postoperative opioid consumption, opioid consumption by POD0-3, and average pain from POD0-3 were compared between groups using Wilcoxon-Mann-Whitney tests. Effect sizes (Cohen’s d, and common language effect size: CL) for these comparisons were also computed. Differences between groups in changes in opioid dose and pain from POD0 through POD3 were analyzed using repeated measures linear mixed models. The focus of those analyses was on the group by POD interaction effect, which tests for differences between groups in the magnitude of changes in opioid dosage or pain over the postoperative study period. Where those interactions were non-significant, we removed them from the models and tested for the main effects of group and day. The sample analyzed is a convenience sample of all available eligible procedures during the designated study period. No prior power analysis was performed.

## Results

We examined 22 patients in the TPP cohort and 20 patients in the ERAS cohort. Patient characteristics for each group are shown in Table 1.

**Table 1:** Patient characteristics *P-values are for between-group comparisons using Wilcoxon-Mann-Whitney tests, except for sex, which used Fisher's exact test. ERAS: enhanced recovery after surgery, TPP: traditional pain pathway

Variable	TPP (N = 22) Mean ± SD	ERAS (N = 20) Mean ± SD	P-value*
Age (y)	14.0 ± 1.8	14.6 ± 1.5	0.52
Sex (% female)	73%	80%	0.72
Height (cm)	163.2 ± 8.4	164.0 ± 8.6	0.72
Weight (kg)	65.8 ± 14.5	59.3 ± 15.9	0.09
Levels (median)	12.0	11.5	0.92
Surgical time (min)	236.9 ± 44.5	232.2 ± 46.9	0.76

Both cohorts had similar age and sex distributions and were of similar height and weight. The median number of levels operated on was nearly identical between cohorts (ERAS: 11.5; TPP: 12.0; P=0.92), and surgical times were similar (ERAS 232.2 min; TPP: 239.6 min; P=0.76).

**Figure 2:**
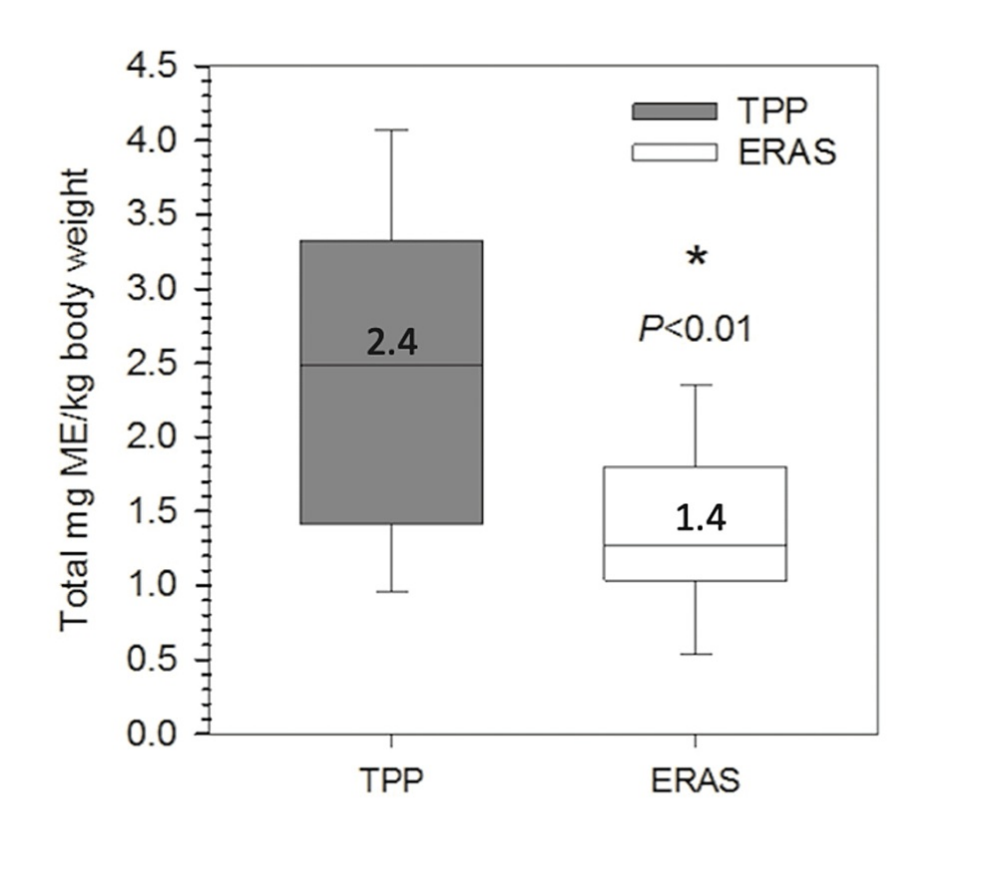
Comparison of the total morphine equivalents per kilogram between the traditional and ERAS pathways. ME/kg: morphine-equivalents per kg, ERAS: enhanced recovery after surgery, TPP: traditional pain pathway

Patients managed with the ERAS pathway experienced a significantly shorter length of hospitalization than the TPP patients. LOS in the ERAS cohort was 1.7 days shorter than in the TPP cohort (ERAS 3.3 + 0.6 days, TPP 5.0 + 1.9 days, P < 0.01), as shown in Figure 2. Within the ERAS group, 100% of patients were discharged by POD5 and 95% stayed four days or less. Of those in the TPP group, 82% were discharged on POD5 or earlier, and four patients required hospitalization until POD7.

When comparing overall opioid consumption between the two groups, we found that ERAS patients required significantly fewer opioids postoperatively. Corrected for weight, total opioid consumption in the ERAS cohort was 1.4 ± 0.7 ME/kg compared to the TPP 2.4 ± 1.1 ME/kg (P < 0.01, Figure 2).

**Figure 3:**
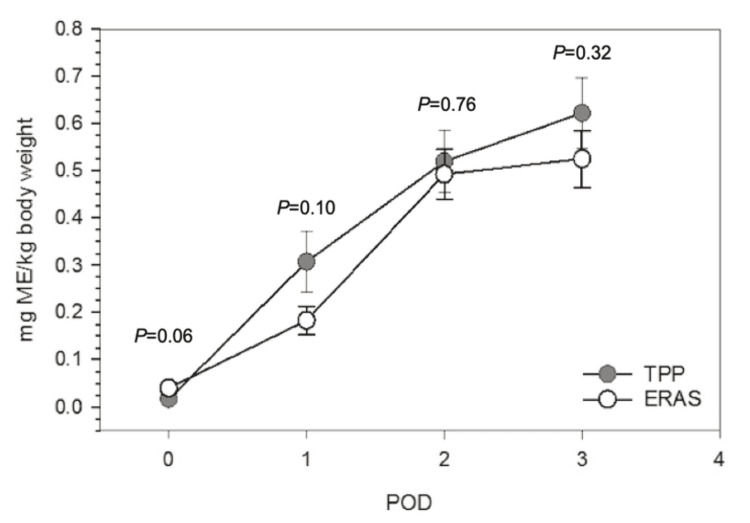
Morphine equivalent per body weight over each postoperative day. TPP: traditional pain pathway, ERAS: enhanced recovery after surgery, POD: postoperative day, ME/kg: morphine-equivalents per kg

**Figure 4:**
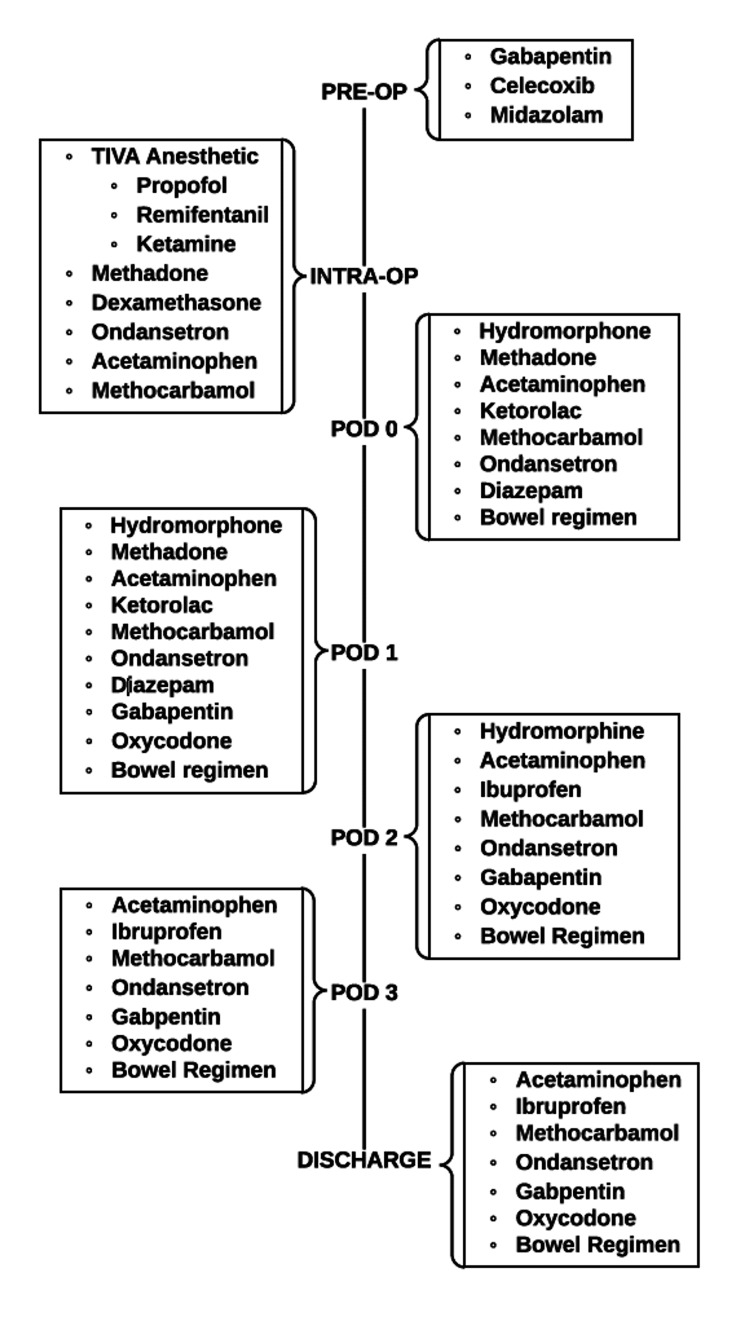
Multimodal ERAS protocol ERAS: enhanced recovery after surgery, POD: postoperative day, TIVA: total intravenous anesthesia

Further analysis compared daily opioid consumption on POD0-3. On POD0, both groups required similar amounts of opioid analgesics (ERAS 0.04 + 0.05 ME/kg, TPP 0.02 + 0.03 ME/kg, P = 0.06). Though not statistically significant, the ERAS group trended towards using fewer opioids when comparing ME/kg on POD 1, 2, and 3 (Figures 3, Table 2).

**Table 2:** Comparison of morphine consumption between groups by day. *P-values are for between-group comparisons using independent samples t-tests ME/kg: morphine-equivalents per kg, ERAS: enhanced recovery after surgery, TPP: traditional pain pathway, POD: postoperative day

	TPP (N = 22)			ERAS (N = 20)			P-value
Variable	Mean	±	SD	Mean	±	SD	
ME/kg POD0	<0.1	±	<0.1	<0.1	±	0.1	0.06
ME/kg POD1	0.3	±	0.3	0.2	±	0.1	0.10
ME/kg POD2	0.5	±	0.3	0.5	±	0.2	0.76
ME/kg POD3	0.6	±	0.4	0.5	±	0.3	0.32
ME/kg Total	2.4	±	1.1	1.4	±	0.7	<0.01

Despite the significant reduction in total opioid consumption by ERAS patients, there was no difference in average VAS pain scores between the two groups (ERAS 3.5 ± 1.4; TPP 2.9 ± 1.2; P=0.15).

Regarding complications, we found that neither group had difficulty with urinary retention or prolonged ventilation. However, the TTP group did have two patients that required a return to surgery. The first developed wound dehiscence and leg weakness that required a repeat trip to surgery and hospitalization for IV antibiotics. The other had a large subfascial hematoma causing sensory changes in their leg, which required a return to surgery for evacuation. Both patients' neurologic deficits returned fully by their first post-operative visit and neither had further wound complications.

## Discussion

The goal of any ERAS pathway is to improve overall patient outcomes using a multimodal, evidenced-based approach [14]. The use of ERAS in adult spine and reconstructive arthroplasty surgeries has benefited patients by delivering shorter hospitalizations, improved pain scores, and substantial cost savings [5]. Implementation of ERAS for our pediatric AIS patients undergoing PSF resulted in a significant reduction in LOS and opioid use while maintaining satisfactory postoperative pain control.

Increased LOS is correlated with morbidity and serves as the surrogate of postoperative milestones. In adolescent scoliosis patients undergoing surgical fixation, prolonged LOS is directly related to increased risk for surgical site infections, sepsis, and readmission [15-17]. In pediatric ERAS protocols, early mobilization is thought to be a contributing factor to decreased LOS and has been shown to improve physical, emotional, and social outcomes in patients [18,19]. Implementation of our ERAS pathway for AIS patients undergoing PSF resulted in a significant reduction in LOS by 1.7 days (p < 0.01) without complications. With discharge criteria at our institution including patients ambulating with minimal assistance, pain adequately controlled with oral pain medication, and the ability to perform activities of daily living independently, we can correlate a reduced LOS to improved patient postoperative outcomes and reduced morbidity.

In addition to enhanced outcomes, ERAS protocols also provide cost savings for patients, their families, and the healthcare system overall [10,20]. PSF for the treatment of AIS is one of the most expensive pediatric surgeries, with charges for a single inpatient POD averaging over $17,000 [21]. Additionally, utilization of an accelerated discharge protocol corresponds to an approximate 22% decrease in postoperative hospital charges after PSF [10]. Implementation of our ERAS protocol resulted in bypassing a PICU admission and a significantly shorter total LOS; therefore, patients presumably also profit from substantial cost savings.

While opioids are frequently prescribed postoperatively, it is not without the potential for significant risk to patients’ health. Short-term complications include acute respiratory depression, constipation, and altered sensorium, while long-term complications include risk for opioid use disorders [22,23]. The adolescent population is at 3-5 times greater risk for serious medical complications related to opioid use compared to younger children, and all of our PSF patients fall into this higher-risk age demographic [23]. Compared to patients using our previous methods for pain control, our ERAS patients demonstrated a significant reduction in opioid consumption by 0.99 ME/kg (P< 0.01). We attribute this reduction to the multimodal nature of our ERAS pathways, leading to more reliance on non-opioid analgesics for postoperative pain control.

Given the accelerated nature of the ERAS pathway with an emphasis on early ambulation, one might anticipate an increase in daily opioid analgesic requirements. We found that the TPP patients used slightly fewer opioids on POD0, but that trend quickly changed to the ERAS group requiring less on POD 1, 2, and 3, as shown in Table 2 and Figure 3.

We attribute the lower opioid consumption on POD0 in the TPP group to the use of intra-operatively administered intrathecal morphine, which was not included in the postoperative opioid utilization calculations. Conversely, the ERAS cohort did not receive intrathecal morphine and instead received postoperative methadone, which was calculated in opioid consumption totals. By removing intrathecal morphine and administering methadone, patients were judged to be at a lower risk for postoperative respiratory depression and therefore able to avoid an overnight stay in the PICU for monitoring.

We must consider the limitations of this study. First, our sample size is relatively small, which is likely due to stringent exclusion criteria. We placed an emphasis on only including healthy patients with adolescent idiopathic scoliosis in order to limit confounding variables. Secondly, the numeric pain scale is fairly subjective and highly variable between patients, and therefore may not be the best way to measure pain. Lastly, since this was an unblinded retrospective study, surgeons were aware of the ERAS pathway implementation and may have introduced bias when discussing expectations with families prior to surgery. Overall, while additional patients would increase the power of the study, we are satisfied that our new ERAS pathway provided significant results.

## Conclusions

The ERAS pathway at our institution significantly reduced LOS and opioid consumption in AIS patients undergoing PSF. We hope this pathway can serve as a model to reduce the overall utilization of opioids for AIS patients, thus reducing the associated risks. Additionally, we feel that this ERAS pathway better controls patients’ post-operative pain allowing for earlier rehabilitation resulting in reduced LOS. While this study only focuses on immediate post-operative care in the hospital setting, further studies would be necessary to assess the effect of the ERAS pathway on recovery as a whole. This could include long-term follow-up with patient-reported outcomes after PSF surgery. Overall, we feel this ERAS pathway has improved recovery after PSF surgery for AIS and continues to be utilized at our institution.
